# Efficacy and Safety of a Single Dose *versus* a Multiple Dose Regimen of Mebendazole against Hookworm Infections in Children: A Randomised, Double-blind Trial

**DOI:** 10.1016/j.eclinm.2018.06.004

**Published:** 2018-07-11

**Authors:** Marta S. Palmeirim, Shaali M. Ame, Said M. Ali, Jan Hattendorf, Jennifer Keiser

**Affiliations:** a Department of Medical Parasitology and Infection Biology, Swiss Tropical and Public Health Institute and University of Basel, Basel, Switzerland; b Public Health Laboratory Ivo de Carneri, Chake Chake, Tanzania; c Department of Epidemiology and Public Health, Swiss Tropical and Public Health Institute and University of Basel, Basel, Switzerland

## Abstract

**Background:**

Single-dose mebendazole is widely used in preventive chemotherapy against the soil-transmitted helminths (STHs) *Ascaris lumbricoides*, hookworm and *Trichuris trichiura*, yet it shows limited efficacy against hookworm and *T. trichiura* infections. The use of adapted treatment regimens might provide a strategy to control and eliminate STH infections in STH-persistent settings. We evaluated the safety and efficacy of the multiple dose mebendazole regimen (3 days 100 mg bid) *versus* a single dose of 500 mg mebendazole in a setting with high STH prevalence and high drug pressure.

**Methods:**

This randomised, double-blind clinical trial took place in a primary school on Pemba Island, Tanzania, in school-aged children (6–12 years). Using a computer random number generator (block size 10), hookworm-positive children were randomly assigned (1:1) to either a single or multiple dose regimen of mebendazole by an independent statistician. Two stool samples were collected at baseline and follow-up (18 to 22 days after treatment) for Kato-Katz analysis. The primary outcome was cure rate (CR) against hookworm. Secondary outcomes were egg reduction rate (ERR) against hookworm, CRs and ERRs against *A. lumbricoides* and *T. trichiura*, and tolerability assessed 3, 24 and 48 h post-treatment. Participants, investigators, caregivers, outcome assessors and the trial statistician were blinded. This trial is registered with ClinicalTrials.gov, number NCT03245398.

**Findings:**

93 children were assigned to each treatment arm. 185 children completed treatment and provided follow-up stool samples. CR against hookworm was significantly higher in the multiple dose (98%) than in the single dose arm (13%, OR 389.1, 95% CI 95.2 to 2885.7%, p < 0.001). 34 and 42 children reported mild adverse events in the single and multiple dose arms, respectively. The most common events were abdominal pain, headache and diarrhoea.

**Interpretation:**

The poor performance of single dose mebendazole against hookworm infections was confirmed, but the multiple dose treatment regimen of mebendazole showed high efficacy. Hence, multiple dose mebendazole might provide a treatment strategy in given epidemiological situations to boost control and elimination of STH infections.

**Funding:**

PATH.

**Research in context**

**Evidence before this study**

We searched in PubMed for all articles published before June 1, 2017 which mentioned both “hookworm” and “mebendazole” in the abstract, with no language restrictions. Although several studies have investigated the effect of either a single or a multiple dose of mebendazole, we only identified one open-label clinical trial, which compared the effect of both the single and the multiple dose mebendazole regimen 16 years ago, prior to commencement of large-scale administration of anthelminthic drugs.

**Added value of this study**

This is the first double-blind randomised clinical trial comparing the effect of a single dose (500 mg) to a multiple dose (100 mg twice a day during three consecutive days) of mebendazole against hookworm infections in Pemba, Tanzania, a setting with high drug pressure and persistent high hookworm prevalence. The results of this study clearly showed that the multiple mebendazole dose is more effective than the single dose. Both regimens were safe with only mild adverse events being reported.

**Implications of all the available evidence**

Currently, the main control strategy against hookworm and other soil-transmitted helminths is preventive chemotherapy, which is based on the administration a single dose of either mebendazole or albendazole. Our study confirms that the curative effect of a single dose mebendazole is not sufficient for treating hookworm infections and that alternative, more effective treatments, as a multiple dose mebendazole regimens might be considered, in particular in persistent hotspot settings.

Alt-text: Unlabelled Box

## 1 Introduction

An estimated 472 million people are infected with hookworms (*Ancylostoma duodenale* and *Necator americanus*). Hookworm disease burden is mainly associated with anaemia, which can cause both physical and intellectual growth retardation among preschool- and school-aged children [1]. In 2016, 1.6 million DALYs were estimated to be caused by hookworm infections [2], leading to annual productivity losses of up to US$139 billion [3].

Currently, the control of hookworm and other soil-transmitted helminths is based on periodic deworming (so called preventive chemotherapy) of school-aged children and other high-risk groups by regularly administering a single dose of either albendazole (400 mg) or mebendazole (500 mg) [4]. Both drugs are highly effective against *Ascaris lumbricoides* but show poor performance against *Trichuris trichiura* when administered as a single dose [5]. Moreover, based on a recent systematic review and network meta-analysis, a single dose of albendazole shows acceptable efficacy against hookworm (CR = 80%), while a single dose of mebendazole fails to clear most of these infections (CR = 33%) [5]. The main anthelmintic drugs available to the control programmes are, therefore, variably efficacious depending on the drug and parasite. Additionally, there are worries that drug resistance will emerge, a problem commonly described in veterinary medicine [6]. With preventive chemotherapy being the predominant tool for helminthiasis control, it might not be surprising that soil-transmitted helminthiasis persists in many settings. As an example, recent studies on Pemba Island reported that prevalence of hookworm continues to range at high levels from 36 to 97% [7], [8], [9], despite regular treatment of school-aged children. Therefore, additional strategies are required to control and eliminate soil-transmitted helminth infections, including access to improved water, sanitation and hygiene [1], as well as adapted treatment regimens, such as optimised dosing or combination chemotherapy which would improve drug therapy.

A multiple dose (100 mg twice per day over three consecutive days) treatment of mebendazole is recommended globally and in Tanzania for individual treatment [10], [11], [12]. However, surprisingly, only a few small studies have evaluated the multiple dose regimen of mebendazole. Moreover, results obtained varied considerably with cure rates (CRs) ranging from 31 to 100% and, therefore, did not allow drawing final treatment recommendations [13], [14], [15]. Finally, only a single study evaluated both treatment arms in an open label trial design, more than 15 years ago [14]. No high quality clinical trial conducted to date did a side-by-side comparison of multiple dose *versus* single dose treatment of mebendazole. Thus, the present trial is, to our knowledge, the first randomised, double-blind trial comparing the efficacy and safety of a single dose (500 mg) to a multiple dose (six doses of 100 mg over three consecutive days twice per day) regimen of mebendazole against hookworm.

## 2 Methods

### 2.1 Study Design and Participants

This randomised, double-blind clinical trial was conducted at Piki Primary School, on Pemba Island, Tanzania, from July 24 to September 15, 2017.

Prior to the study initiation, ethical approval was obtained from the Zanzibar Medical Research and Ethical Committee (ZAMREC, reference number 0002/May/17) and from the Ethics Committee of Northern and Central Switzerland (EKNZ, reference number 2017-00320). This trial is registered with ClinicalTrials.gov, number NCT03245398.

Before enrolment, all caregivers of children aged 6–12 years attending the primary school of Piki village were invited to information sessions at school during which the research staff explained the purpose and procedures of the study, as well as the benefits and potential risks of participating. Caregivers had the chance to clarify any questions they may had before they were asked whether they wanted their child to be included in the study or not. Caregivers who chose to allow the participation of their child were asked to sign a written informed consent. Illiterate caregivers provided a thumbprint while an impartial witness signed to verify that all information in the informed consent form was conveyed appropriately.

Consenting children were eligible if they had provided two stool samples, were positive for hookworm eggs in the stool (≥ 100 eggs per gram [EPG] or at least two Kato-Katz thick smears slides with more than one hookworm egg). After the initial clinical examination, children were excluded from the trial if any of the following exclusion criteria were present: had menarche (for females); presence of severe anaemia (haemoglobin < 8.0 g/dl was considered severe anaemia); had any known or reported history of chronic illness such as cancer, diabetes, chronic heart, liver or renal disease; received any recent anthelminthic treatment (within past 4 weeks); had any known allergy to mebendazole or albendazole.

### 2.2 Randomisation and Masking

The trial statistician (JH), who was not involved in any field work, provided a computer-generated stratified (by baseline infection intensities) block randomisation code (blocks of size ten). Participants were allocated 1:1 to one of the two treatment arms: single dose (500 mg) or multiple dose (100 mg twice a day during three consecutive days) of mebendazole. 500 mg and 100 mg mebendazole tablets were obtained from Johnson & Johnson (Zug, Switzerland). Matching placebos were manufactured at the University of Basel (100 mg mebendazole matching placebo) or purchased at Fagron, Germany (500 mg mebendazole matching placebo). Trial medications were prepared in advance in identical plastic envelopes labelled with the children's unique treatment identification numbers and sealed. The treatment lasted 3 days and children received tablets at six different time points (mornings and evenings of each of the 3 days). At the first time point, all participants received two tablets: either 500 mg mebendazole plus 100 mg mebendazole matching placebo, or 500 mg mebendazole matching placebo and 100 mg mebendazole; at the remaining five time points, children only received one tablet: either 100 mg mebendazole or matching placebo, depending to which treatment arm they were allocated. Participants, investigators, caregivers, outcome assessors and the trial statistician were blinded. Allocation was concealed: the envelopes containing the drugs were in bags of ten and, within each group of ten, envelopes were stacked on each other, preventing the administrator from seeing the next envelope.

### 2.3 Study Procedures

The name, age, sex and school grade of eligible children were recorded. Children received containers labelled with their unique identification number (ID) and were asked to provide two stool samples, preferably on consecutive days. Stool samples were transferred to the Public Health Laboratory Ivo de Carneri where duplicate Kato-Katz thick smear slides were prepared from each sample [16]. Slides were examined by experienced laboratory technicians under a light microscope within 1 h after slide preparation, to avoid clearing of hookworm eggs. Helminth eggs were enumerated and recorded for each parasite (hookworm, *A. lumbricoides* and *T. trichiura*) separately. To ensure quality of hookworm diagnosis, 10% of the samples were divided into two stool containers; one of the containers kept its original participant ID, whereas the second container was labelled with a new ID (assigned by the co-investigator). Duplicate Kato-Katz were prepared from both containers and the findings compared. Quality control for *A. lumbricoides* and *T. trichiura* consisted of re-reading 10% of all slides. Any discrepancies between the original and the quality control read were discussed [17].

All children found positive for hookworm underwent a physical and clinical examination by a physician. Height was measured with a standard meter (to the nearest 0.1 cm), weight with an electronic balance (to the nearest 0.1 kg), and temperature using an electronic ear thermometer (to the nearest 0.1 °C). Haemoglobin levels were measured in capillary blood using the finger-prick method (HemoCue® 301). Children were examined for any acute or chronic illness and questioned about their medical history.

Eligible children were enrolled (by MSP) for treatment which took place at Piki Primary school during the 3 days following the physical and clinical examination. Children received treatment with clean water and a package of biscuits. After every morning treatment children were monitored for 3 h and then actively questioned for adverse events by the study physician and nurses using a questionnaire. The same procedure took place 24 and 48 h after every morning treatment. At follow-up, between 18 to 22 days after treatment, another two stool samples were collected from each treated child. Participants who remained infected with hookworm, *A. lumbricoides* and/or *T. trichiura* at the end of the study were treated with albendazole (400 mg). Similarly, children who did not fulfil the eligibility criteria but were found infected with at least one of the parasites during screening were treated with albendazole.

### 2.4 Outcomes

The primary outcome of this study was the CR against hookworm 20 days (± 3 days) after treatment using the Kato-Katz thick smear method. CR was defined as percentage of hookworm-positive children being negative at follow-up. Secondary outcomes were (i) egg reduction rate (ERR) against hookworm, (ii) CR and ERR against *A. lumbricoides* and *T. trichiura*, and (iii) tolerability (number of adverse events) assessed 3, 24 and 48 h post-treatment.

An additional secondary outcome, which will not be addressed in the current manuscript, was caregiver's knowledge related to the clinical trial after attending an information session. This outcome was assessed using a short questionnaire.

### 2.5 Sample Size

A CR of 20% was assumed for the single dose mebendazole [9] and a CR of 40% was assumed for the multiple dose treatment regimen [18], [19], [20]. To detect a difference with 80% power at a two-sided 5% significance level, a minimum of 79 participants per study arm was required. Accounting for a loss to follow-up of 12%, we obtained a final sample size of 180 participants (90 per arm).

### 2.6 Statistical Analysis

Data were double entered by two staff members into a database (Access 2003, Microsoft) and crosschecked using the Data Compare tool of EpiInfo version 3.3.2. Any discrepancies between both data entries were resolved by consulting the original records. Statistical analyses were performed using STATA 14.0 (StataCorp) and R 3.4.3 (R Development Core Team). Only children who submitted samples before and after treatment were included in the available case analysis, which followed intention to treat principles. Odds ratios (OR) were calculated using unadjusted (primary analysis) and adjusted logistic regression (adjustment for age, sex, weight and strata). For ERR calculation, the mean egg count of the four Kato-Katz thick smears was multiplied by a factor of 24 to calculate the EPG. ERR was defined as the percentage of mean reduction at follow-up compared to baseline. ERR was calculated using both the geometric mean (GM) and the arithmetic mean (AM). Confidence intervals for ERRs were calculated using the bootstrap resampling method with 5000 replicates. The significance level was set at p-value ≤ 0.05. Infection intensities (light, moderate or heavy) were determined according to WHO cut-offs [21].

### 2.7 Role of Funding Source

The funder of the study had input into the study design, but no role in data collection, data analysis, data interpretation, or writing of the report. The corresponding author had full access to all the data in the study and had final responsibility for the decision to submit for publication.

## 3 Results

**Fig. 1:**
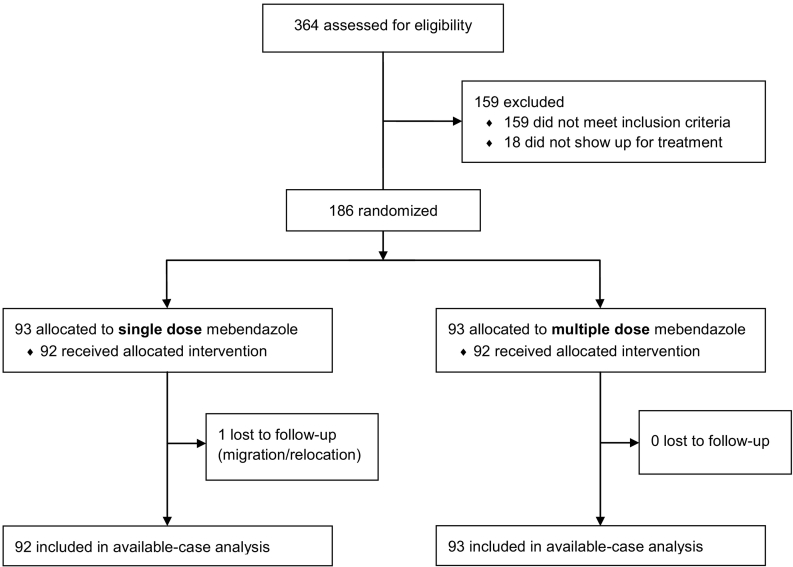
Trial profile.

364 consenting participants were screened for hookworm. 354 children provided two baseline stool samples. Of these, 206 were found hookworm positive and were invited for the clinical and physical examination. Two children were excluded because they had haemoglobin levels below 8.0 g/dl and 18 were absent from school on the clinical and physical examination day. The remaining 186 children were present for treatment and 93 participants were randomly allocated to each treatment arm. One child from the single dose of mebendazole arm was lost to follow-up because he/she was not on Pemba Island during the follow-up period. With the exception of 11 children, who provided the second baseline stool sample between six and 19 days after the first sample, both stool samples from all other children were collected within a five-day window. All remaining 185 participants who provided follow-up stool samples were included in the available case analysis (Fig. 1).

At the second treatment time point, two children's envelopes were swapped. Therefore, by the end of the six-treatment time points, one child had erroneously received 100 mg of mebendazole in addition to the single dose of 500 mg of mebendazole plus placebo and the other child received only five doses instead of six doses of 100 mg of mebendazole plus 100 mg of placebo. These two subjects were included in the available case analysis but excluded from the per protocol analysis (n = 183) (Appendices 1 and 2). Appendix 3 presents the results of the analysis using the intention-to-treat population (n = 186).

The treatment arms were balanced according to age, sex, weight, height, and hookworm baseline infection intensity (Table 1). During the physical examination, three children were found to have *Tinea capitis*, one child reported having asthma and one sickle cell anaemia. These children were not excluded.

**Table 1:** Baseline characteristics of all randomised children stratified by treatment arm. Data are n (%), median (IQR), mean (SD). BMI = body-mass index, EPG = eggs per gram of stool.

	Single dose (n = 93)	Multiple dose (n = 93)
Mean age (years)	10.1 (1.6)	10.1 (1.6)
Girls	39 (42%)	46 (50%)
Mean weight (kg)	26.7 (5.3)	26.2 (5.1)
Mean height (cm)	132.0 (10.2)	131.8 (9.6)
Mean weight-for-age Z-score	− 1.4 (1.2)	− 1.3 (0.8)
Mean height-for-age Z-score	− 1.1 (1.1)	− 1.1 (0.7)
Mean BMI-for-age Z-score	− 1.1 (0.9)	− 1.2 (0.8)
Hookworm		
Infected children	93 (100%)	93 (100%)
Median	222 (78-534)	222 (96-606)
EPG geometric mean	219.0	234.2
Infection intensity		
Light (1–1999 EPG)	89 (96%)	90 (97%)
Moderate (2000–3999 EPG)	3 (3%)	3 (3%)
Heavy (≥ 4000 EPG)	1 (2%)	0
*Trichuris trichiura*		
Infected children	88 (96%)	91 (98%)
EPG geometric mean	661.8	725.7
Infection intensity		
Light (1–999 EPG)	59 (66%)	56 (62%)
Moderate (1000–9999 EPG)	30 (34%)	35 (38%)
Heavy (≥ 10,000 EPG)	0	0
*Ascaris lumbricoides*		
Infected children	47 (51%)	51 (55%)
EPG geometric mean	2691.2	4095.9
Infection intensity		
Light (1–4999 EPG)	28 (60%)	20 (39%)
Moderate (5000–49,999 EPG)	15 (32%)	28 (55%)
Heavy (≥ 50,000 EPG)	4 (8%)	3 (6%)

At baseline, among the 354 children who provided two stool samples, 94.3% of children were infected with at least one soil-transmitted helminth and 29.4% were co-infected with all three soil-transmitted helminths. The prevalences of hookworm, *A. lumbricoides* and *T. trichiura* were 58.2, 36.7 and 92.6%, respectively. In terms of intensity of infections, 4% of hookworm, 31% of *T. trichiura* and 45% of *A. lumbricoides* infections were moderate or heavy.

The CR of the multiple dose of mebendazole against hookworm was significantly higher (CR = 97.9%) than that of the single dose (CR = 13.0%, Odds ratio [OR] 303.3, 95% confidence interval [CI] 81.6 to 1999.4, p < 0.001). Superiority was confirmed by the adjusted logistic regression model (CR = 13.0% *versus* CR = 97.8%, OR 389.1, 95% CI 95.2 to 2885.7%, p < 0.001). In terms of ERR, the multiple dose (GM ERR = 100.0%) was also significantly more effective than the single dose (GM ERR = 68.0%) (Difference = − 0.32, 95% CI − 0.46 to − 0.22) (Table 2). ERRs obtained with the arithmetic mean were 99.8% versus 52.7%.

**Table 2:** Cure rates (CRs) and egg reduction rates (ERRs) against hookworm, *Ascaris lumbricoides* and *Trichuris trichiura* after the administration of a single or multiple doses of mebendazole. CR = cure rate, CI = confidence interval, EPG = eggs per gram of stool, ERR = egg reduction rate.

	Single dose	Multiple dose
Hookworm		
Children positive before treatment	92	93
Children cured after treatment	12	91
CR (95% CI)	13.0% (6.9–21.7)	97.9% (92.4–99.7)
EPG geometric mean		
Before treatment	220.2	234.9
After treatment	70.5	0.1
ERR (95% CI)	68.0% (51.5–78.6)	100% (99.9–100)
EPG arithmetic mean		
Before treatment	442.6	465.3
After treatment	209.3	1
ERR (95% CI)	52.7% (40.3–63.6)	99.8% (99.3–100)
*Trichuris trichiura*		
Children positive before treatment	88	91
Children cured after treatment	6	39
CR (95% CI)	6.8% (4.6–17.8)	42.9% (33.8–54.8)
EPG geometric mean		
Before treatment	655.9	726.8
After treatment	185.5	14.0
ERR (95% CI)	71.7% (56.7–78.5)	98.1% (96.8–98.7)
EPG arithmetic mean		
Before treatment	1017.4	1263.8
After treatment	517.6	105.8
ERR (95% CI)	49.1% (31.7–61.0)	91.6% (88.4–94.6)
*Ascaris lumbricoides*		
Children positive before treatment	47	51
Children cured after treatment	47	50
CR (95% CI)	100.0%	98.0% (94.2–100)
EPG geometric mean		
Before treatment	2698.5	4113
After treatment	0	0.2
ERR (95% CI)	100.0%	100%
EPG arithmetic mean		
Before treatment	14,597.5	14,859.9
After treatment	0	130.9
ERR (95% CI)	100.0%	99.1% (96.9–100)

42.9% of children were cured against *T. trichiura* following six doses of mebendazole, compared to 6.8% in the single dose arm; this difference was statistically significant (OR 42.9, 95% CI 4.3 to 28.5, p < 0.001 with the unadjusted model; OR 13.4, 95% CI 5.4 to 39.6, p < 0.001 with the adjusted model). GM ERRs against *T. trichiura* were 71.7% (95% CI 56.7–78.5) in the single and 98.1% (95% CI 96.8–98.7) in the multiple dose arm. The corresponding ERRs based on AM were 49.1% and 91.6%, respectively. The single dose of mebendazole cured all children with an *A. lumbricoides* infection and the multiple dose cured all but one child (Table 2). In both arms the ERRs against *A. lumbricoides* were > 99.9%. Table 3 shows that among the 65 children with moderate *T. trichiura* infections at baseline, 10 were cured (all in the multiple dose arm), 43 turned from moderate into light infections (19 in the single dose arm and 24 in the multiple dose arm), and 11 children remained at moderate infection intensity (10 in the single and one in the multiple dose arm).

**Table 3:**
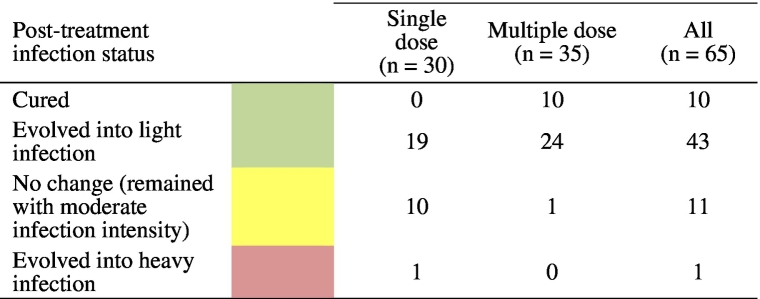
Number of children with moderate *T. trichiura* infections at baseline which, post-treatment, were either cured, evolved into light or heavy infections, or remained with moderate infection intensity.

**Fig. 2:**
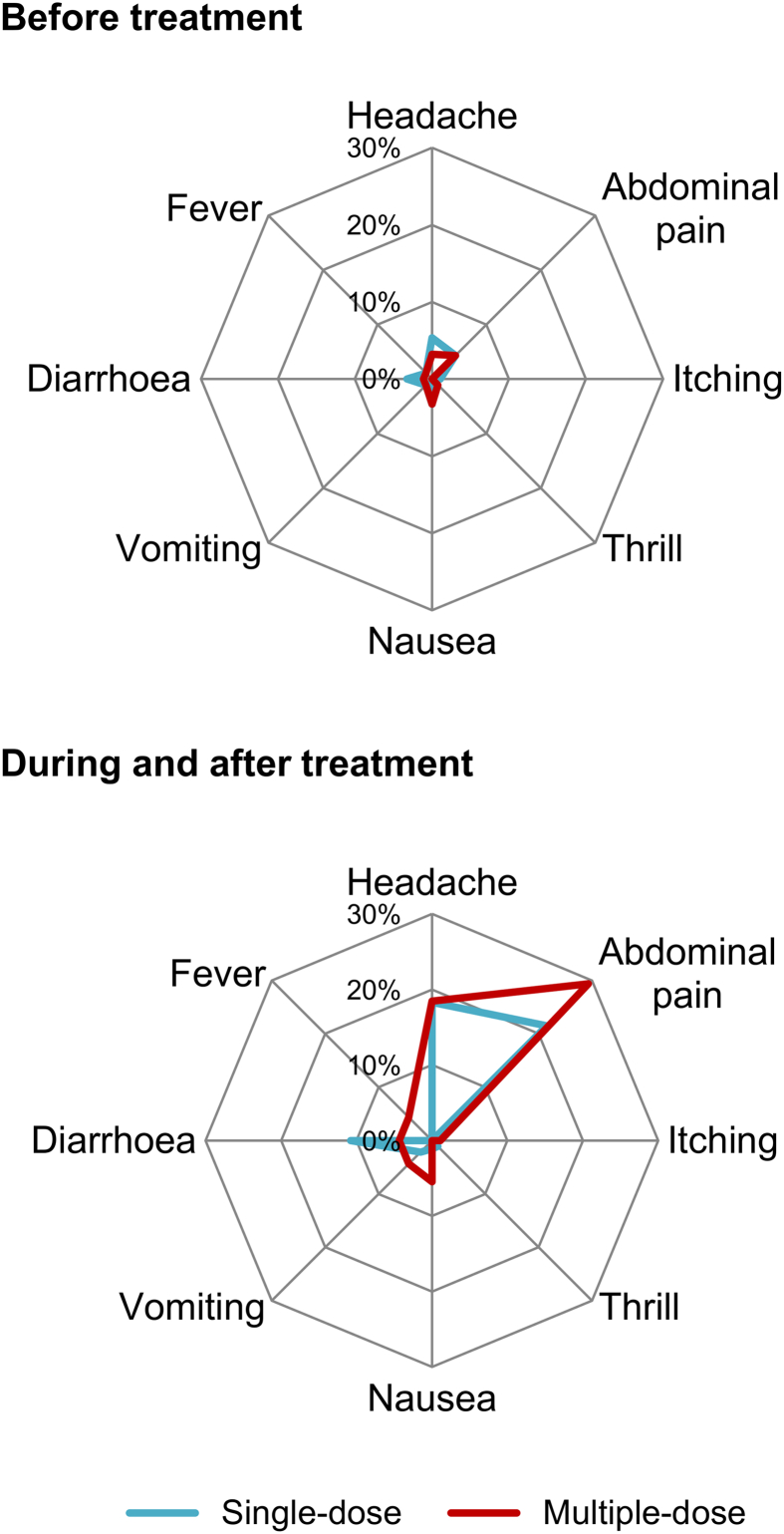
Spider plot indicating the percentage of observed clinical symptoms (before treatment) and adverse events in both treatment arms (throughout all seven adverse event assessment time points).

At the clinical examination, which took place right before treatment, a total of 20 children (11.3%) reported symptoms; 10 in the single dose arm and 10 in the multiple mebendazole dose arm (Appendix 4). The number of adverse events and children reporting adverse events stratified by treatment arm and evaluation time point are summarised in Table 4. Children in the multiple dose treatment arm reported slightly more adverse events than those in the single dose arm. In total, throughout all adverse event assessment time points, 34 children (37%) in the single treatment arm reported symptoms and in the multiple arm 42 children (45%) reported symptoms after treatment (Appendix 4). The most commonly reported adverse events were abdominal pain (69 reports), headache (46 reports) and diarrhoea (17 reports) during all treatment points. All events were mild. A visual examination of the number of children reporting each type of adverse event throughout the whole treatment is available in Fig. 2.

**Table 4:** Number of symptoms reported during the clinical examination and number of children reporting the symptoms during the clinical examination; number of adverse events (AEs) reported by children and number of children reporting AEs at each of the AE assessment time points by treatment arm.

Timepoint	Number of	Single dose (n = 93)	Multiple dose (n = 93)	Total
Clinical examination before treatment	Symptoms	17	13	30
	Children	9	9	18
3 h after 1st morning treatment	AEs	10	13	23
	Children	10	11	21
24 h after 1st morning treatment	AEs	4	4	8
	Children	4	4	8
3 h after 2nd morning treatment	AEs	12	12	24
	Children	6	10	16
24 h after 2nd morning treatment	AEs	11	13	24
	Children	9	9	18
3 h after 3rd morning treatment	AEs	7	12	19
	Children	5	10	15
24 h after 3rd morning treatment	AEs	9	6	15
	Children	7	5	12
48 h after 3rd morning treatment	AEs	21	28	49
	Children	17	22	39
Total during all AE assessment time points	AEs	91	101	192
	Children	34	42	76

## 4 Discussion

Preventive chemotherapy is the mainstay of helminthiases control, since it remains among the most cost-effective global public health control measures [22]. Albendazole and mebendazole, which are variably efficacious against the different soil-transmitted helminths, are the most widely used drugs. We were interested in learning whether treatment efficacy could be improved by an adapted treatment regimen in a setting such as Pemba Island which, even though community members have been receiving treatment once or twice a year for 25 years [23], is still characterised by intense helminth transmission and persisting high prevalence rates [9]. Using a double-blind trial design we evaluated the multiple dose (3 day, 6 dose course) treatment of mebendazole, which is recommended globally and in Tanzania for individual treatment [10], [11], [12], [24], *versus* the single dose regimen widely used for population-based treatment.

We found clear evidence that the multiple, six-dose treatment schedule of mebendazole is significantly more effective at curing hookworm infections than a single dose of mebendazole. In our trial, only 13% of children were cured after a 500 mg single dose of mebendazole. On the other hand, the multiple dose regimen of mebendazole cured almost all hookworm-infected children (CR = 98%) which is in agreement with exploratory studies in the early 1970s [25]. To our knowledge, only four RCTs, conducted in Iran, Thailand, Brazil and Papua New Guinea, assessed the effect of the multiple dose mebendazole on hookworm infections [14], [18], [19], [20]. In these studies, CRs ranged from 35 to 94%. Although these studies reported different baseline infection intensities, there seems to be no correlation between the intensity of infection and CRs.

Concerns have been raised that mebendazole resistance had developed in the setting of Pemba since reduced efficacy of this drug was observed. In more detail, treatment of hookworm infections resulted in significantly lower cure (7.6%) and ERR (52.1%) in 2003 than the ones reported before the beginning of periodic chemotherapy (CR = 22.4%, ERR = 82.4%) [26]. However, our findings clearly demonstrate that with using the recommended treatment regimen (which does not include dose intensification or dose density of chemotherapy, strategies commonly employed for example in the treatment of cancer [27]) for mebendazole, high efficacy against hookworm can still be obtained and that speculations on drug resistance of mebendazole against hookworm should therefore be considered with caution. On the other hand, it is interesting to note that the above mentioned strategies of dose intensification did not result in higher efficacy of mebendazole against soil-transmitted helminth infections. For example, RCTs evaluating the effect of 500 mg of mebendazole daily for 3 days (1500 mg in total) found CRs ranging of 26 to 59% [28], [29]. Thus, the higher dose does not seem to be the most important determinant driving the drug's effect.

Overall, the hookworm CR for single dose mebendazole we observed on Pemba Island (13%) is in line with results from RCTs conducted in the same setting [9], [26], however is considerably lower than a CR of 33% calculated from 13 trials in a recent meta-analysis [5]. This discrepancy between our results and the summary estimate by means of meta-analysis could be due to several factors such as the diagnostic method used, the parasite strain, or the study location: three of the four RCTs which reported CRs below 20% following a single dose of mebendazole against hookworm took place on Pemba Island. Another influencing factor could be study quality and the sample size: the two RCTs reporting highest CRs using the single dose had small sample sizes of 35 and 45 participants in the single dose mebendazole arms [24], [30].

Our study showed that the multiple dose of mebendazole was also considerably more effective against *T. trichiura* infections than the single 500 mg dose. Not only CRs were higher (CR = 42.9% *versus* CR = 6.8% respectively) but moderate infection intensities were less commonly observed in the multiple dose arm. Our results for the multiple dose regimen against *T. trichiura* are in line with previous studies conducted in Papua New Guinea and Brazil (CR = 65% and CR = 39%). However, similarly to what we found for the efficacy on hookworm, our results for the single dose were markedly lower than summary estimates reported by Moser and colleagues (CR = 42%) [5].

Unlike the other two parasites, a multiple dose of mebendazole does not present an advantage against *A. lumbricoides* infections over a single mebendazole dose.

Overall, both mebendazole treatments were well tolerated. Interestingly, available data on adverse events following multiple mebendazole doses is sparse. None of the three RCTs which tested the efficacy of the multiple dose mebendazole regimen documented information on adverse events. Headache, abdominal pain or diarrhoea were most commonly reported in both treatment arms, which is in line with previous studies exploring the efficacy and safety of 500 mg mebendazole [24], [31]. In the current study, we found a sudden increase in the number of adverse events 48 h after the last treatment which is rather unexpected due to the short half-life of mebendazole (in the range of 2–9 h) [32]. For comparison, Speich *et al.* assessed adverse events at 3, 24, 27 and 48 h post-treatment following a single dose of mebendazole and found the peak number of symptoms at 24 h [9]. It is important to highlight that the same trend was observed in both treatment arms (hence 48 h and 96 h after the last active treatment), which suggests that this finding might not be triggered by the treatment but rather by differences in the reporting.

One limitation of this study is that the Kato-Katz technique has low sensitivity, particularly for light infections [17]. As an effort to overcome this limitation, two stool samples were collected on different days and two slides were prepared from each sample. However, this may still result in an overestimation of CRs as light infections at follow-up might have been missed and falsely considered as cured. Additionally, the collection of two follow-up samples from 11 children was spaced by more than 5 days. Although there is no literature on the issue of how many days between sample collection are recommendable, this could had some impact on the outcome.

In conclusion, our study has shown that the multiple dose regimen of mebendazole is unarguably more effective against hookworm and concomitant *T. trichiura* infections. The findings of our study add to recent results demonstrating that adapted treatments, including improved dosages, regimen or drug combinations considerably increase and broaden the spectrum of activity against soil-transmitted helminth infections. A multiple dose regimen clearly involves more resources than the administration of a single dose or drug combinations and comes with additional logistic challenges. However, in hotspot settings such as Pemba Island where the prevalences of hookworm and *T. trichiura* are so high despite decades of treatment, this could be a strategy to consider. In the framework of preventive chemotherapy, drugs are distributed by non-medical personnel (such as teachers, volunteers or community drug distributors) in non-medical settings such as schools [33]. Thus, although more challenging, teachers could provide six doses instead of a single dose of mebendazole to each school-child. Improved treatments would trigger a considerable decrease of infections which would lead to a reduction of reservoirs that sustain reinfections in the population. In parallel, efforts should continue to discover and develop novel drugs and vaccines, which n the long-term would aid in the elimination of these diseases [34].

## Contributors

MSP, Said MA, Shaali MA, JH, and JK planned and designed the study; MSP, Said MA, Shaali MA, and JK conducted the study; MSP, JH and JK analysed and interpreted the trial data; MSP and JK wrote the first draft and JH revised the manuscript. All authors read and approved the final version of the manuscript.

## Appendix A Supplementary data

Supplementary material 1 Image 1

Supplementary material 2 Image 2
