# Supplementary material for: Efficacy and Safety of a Single Dose versus a Multiple Dose Regimen of Mebendazole against Hookworm Infections in Children: A Randomised, Double-blind Trial
Source: eClinicalMedicine. 2018 Jul 11;1:7–13. doi: 10.1016/j.eclinm.2018.06.004 (PMC6537524; doi:10.1016/j.eclinm.2018.06.004)
Supplement: Supplementary file 1 — Supplementary material 1 [file mmc1.docx]

**Supplementary Appendix**

**Table of contents**

Authors and institutional affiliations……………………………………………………………………..2

Table S1 – Baseline characteristics (per protocol) ………….…...……………………….………….3

Table S2 – CRs and ERRs (per protocol) …...………………………………………………...……...4

Table S3 – CRs and ERRs (intention-to-treat) …...…………………………………………...……...5

Table S4 – Types of symptoms ………….…...………………………………………………...……...6

Table S5 – Number of symptoms ………..…...………………………………………………...……...7

**Efficacy and safety of a single dose *versus* a multiple dose regimen of mebendazole against hookworm infections in children: a randomised, double-blind trial**

Marta S. Palmeirim, Shaali M. Ame, Said M. Ali, Jan Hattendorf, Jennifer Keiser

**Department of Medical Parasitology and Infection Biology, Swiss Tropical and Public Health Institute, and University of Basel, Basel, Switzerland** (MS Palmeirim PhD, Prof J Keiser PhD); **Public Health Laboratory Ivo de Carneri, Chake Chake, Tanzania** (SM Ali, SM Ame); **Department of Epidemiology and Public Health, Swiss Tropical and Public Health Institute, and University of Basel, Basel, Switzerland** (J Hattendorf PhD)

**Table S1. Baseline characteristics of children following per protocol analysis stratified by treatment arm.** Data are n (%), median (IQR), mean (SD). EPG = eggs per gram of stool.

|  | | | Single dose (n=91) | Multiple dose (n=92) |  |  |
| --- | --- | --- | --- | --- | --- | --- |
| Mean age (years) | | | 10∙2 (1∙6) | 10∙1 (1∙6) |  |  |
| Girls | | | 37 (41%) | 45 (49%) |  |  |
| Mean weight (kg) | | | 26∙6 (5∙3) | 26∙1 (5∙1) |  |  |
| Mean height (cm) | | | 132∙0 (10∙3) | 131∙9 (9∙7) |  |  |
| Hookworm | | |  |  |  |  |
|  | Infected children | | 91 (100%) | 92 (100%) |  |  |
|  | EPG geometric mean | | 222.9 | 231.7 |  |  |
|  | Infection intensity | |  |  |  |  |
|  |  | Light (1-1999 EPG) | 87 (96%) | 89 (97%) |  |  |
|  |  | Moderate (2000-3999 EPG) | 3 (3%) | 3 (3%) |  |  |
|  |  | Heavy (≥4000 EPG) | 1 (2%) | 0 |  |  |
| *Trichuris trichiura* | | | | |  |  |
|  | Infected children | | 87 (96%) | 90 (98%) |  |  |
|  | EPG geometric mean | | 664.2 | 720.3 |  |  |
|  | Infection intensity | |  |  |  |  |
|  |  | Light (1-999 EPG) | 58 (67%) | 56 (62%) |  |  |
|  |  | Moderate (1000-9999 EPG) | 29 (33%) | 34 (38%) |  |  |
|  |  | Heavy (≥10000 EPG) | 0 | 0 |  |  |
| *Ascaris lumbricoides* | | |  |  |  |  |
|  | Infected children | | 46 (51%) | 51 (55%) |  |  |
|  | EPG geometric mean | | 2987.7 | 4113 |  |  |
|  | Infection intensity | |  |  |  |  |
|  |  | Light (1-4999 EPG) | 27 (58%) | 20 (39%) |  |  |
|  |  | Moderate (5000-49999 EPG) | 15 (33%) | 28 (55%) |  |  |
|  |  | Heavy (≥50000 EPG) | 4 (9%) | 3 (6%) |  |  |

**Table S2. Cure rates (CRs) and egg reduction rates (ERRs) of hookworm, *Ascaris lumbricoides* and *Trichuris trichiura* after the administration of a single or multiple dose of mebendazole of the per protocol population.** GM = geometric mean; AM = arithmetic mean; ERR = egg reduction rate.

|  | | Single dose | Multiple dose |
| --- | --- | --- | --- |
| **Hookworm** | |  |  |
|  | Children positive before treatment | 91 | 92 |
|  | Children cured after treatment | 12 | 91 |
|  | CR | 13∙2% | 97∙8% |
|  | GM ERR | 68∙4% | 100% |
|  | AM ERR | 52∙8% | 99∙8% |
| ***Trichuris trichiura*** | |  |  |
|  | Children positive before treatment | 87 | 90 |
|  | Children cured after treatment | 6 | 39 |
|  | CR | 6∙9% | 43∙3% |
|  | GM ERR | 71∙8% | 98∙1% |
|  | AM ERR | 49∙1% | 91∙6% |
| ***Ascaris lumbricoides*** | |  |  |
|  | Children positive before treatment | 46 | 51 |
|  | Children cured after treatment | 46 | 50 |
|  | CR | 100∙0% | 98∙0% |
|  | GM ERR | 100∙0% | 100% |
|  | AM ERR | 100∙0% | 99∙1% |

**Table S3. Cure rates (CRs) and egg reduction rates (ERRs) of hookworm, *Ascaris lumbricoides* and *Trichuris trichiura* after the administration of a single or multiple dose of mebendazole of the intention-to-treat population.** GM = geometric mean; AM = arithmetic mean; ERR = egg reduction rate.

|  | | Single dose | Multiple dose |
| --- | --- | --- | --- |
| **Hookworm** | |  |  |
|  | Children positive before treatment | 93 | 93 |
|  | Children cured after treatment | 12 | 91 |
|  | CR | 12.9% | 97.9% |
|  | GM ERR | 68.0% | 99.9% |
|  | AM ERR | 52.7% | 99.8% |
| ***Trichuris trichiura*** | |  |  |
|  | Children positive before treatment | 89 | 91 |
|  | Children cured after treatment | 5 | 39 |
|  | CR | 5.6% | 42.9% |
|  | GM ERR | 71.7% | 98.1% |
|  | AM ERR | 49.1% | 91.6% |
| ***Ascaris lumbricoides*** | |  |  |
|  | Children positive before treatment | 47 | 51 |
|  | Children cured after treatment | 47 | 50 |
|  | CR | 100% | 98.0% |
|  | GM ERR | 100% | 100% |
|  | AM ERR | 100% | 99.2% |
|  |  |  |  |

**Table S4. Number of children reporting symptoms before treatment, number of children reporting adverse events during and after treatment, and number of adverse event episodes reported during and after treatment by arm (n=93 in each arm).**

|  | **Number of children with symptoms before treatment** | | **Number of children with adverse events** | | **Total number of adverse event episodes** | |
| --- | --- | --- | --- | --- | --- | --- |
| **Symptom** | **Single** | **Multiple** | **Single** | **Multiple** | **Single** | **Multiple** |
| Headache | 5 (5%) | 3 (3%) | 17 (18%) | 17 (18%) | 25 | 21 |
| Abdominal pain | 4 (4%) | 4 (4%) | 20 (22%) | 27 (29%) | 27 | 42 |
| Itching | 1 (1%) | 0 | 0 | 1 (1%) | 0 | 1 |
| Thrill | 0 | 1 (1%) | 1 (1%) | 0 | 1 | 0 |
| Nausea | 3 (3%) | 3 (3%) | 1 (1%) | 5 (6%) | 1 | 7 |
| Vomiting | 1 (1%) | 1 (1%) | 2 (2%) | 4 (4%) | 2 | 5 |
| Diarrhoea | 3 (3%) | 1 (1%) | 10 (11%) | 4 (4%) | 12 | 5 |
| Fever | 1 (1%) | 1 (1%) | 0 | 4 (4%) | 0 | 3 |
| Any symptom | 10 (11%) | 10 (11%) | 34 (37%) | 42 (45%) |  |  |

**Table S5. Number of symptoms reported before, during and after treatment at the different adverse event assessment time points by treatment arm.**

| **Time point** | **Symptom** | **Single dose** | **Multiple dose** | **Total** |
| --- | --- | --- | --- | --- |
| Clinical examination  (before treatment) | Headache | 5 | 3 | 8 |
|  | Abdominal pain | 4 | 4 | 8 |
|  | Itching | 1 | 0 | 1 |
|  | Thrill | 0 | 1 | 1 |
|  | Nausea | 3 | 3 | 6 |
|  | Vomiting | 1 | 1 | 2 |
|  | Diarrhoea | 3 | 1 | 4 |
|  | Fever (≥ 37.5 ºC) | 1 | 1 | 2 |
|  | Other | 0 | 0 | 0 |
| 3h after the first treatment | Headache | 6 | 2 | 8 |
|  | Abdominal pain | 2 | 8 | 10 |
|  | Itching | 0 | 0 | 0 |
|  | Thrill | 0 | 0 | 0 |
|  | Nausea | 1 | 2 | 3 |
|  | Vomiting | 0 | 1 | 1 |
|  | Diarrhoea | 1 | 0 | 1 |
|  | Fever (≥ 37.5 ºC) | 0 | 0 | 0 |
|  | Other | 0 | 0 | 0 |
| 24h after the first treatment | Headache | 1 | 0 | 1 |
|  | Abdominal pain | 3 | 3 | 6 |
|  | Itching | 0 | 0 | 0 |
|  | Thrill | 0 | 0 | 0 |
|  | Nausea | 0 | 0 | 0 |
|  | Vomiting | 0 | 1 | 1 |
|  | Diarrhoea | 0 | 0 | 0 |
|  | Fever (≥ 37.5 ºC) | 0 | 0 | 0 |
|  | Other | 0 | 0 | 0 |
| 3h after the second treatment | Headache | 3 | 3 | 6 |
|  | Abdominal pain | 3 | 7 | 10 |
|  | Itching | 0 | 0 | 0 |
|  | Thrill | 1 | 0 | 1 |
|  | Nausea | 0 | 0 | 0 |
|  | Vomiting | 1 | 1 | 2 |
|  | Diarrhoea | 2 | 0 | 2 |
|  | Fever (≥ 37.5 ºC) | 0 | 0 | 0 |
|  | Other | 0 | 0 | 0 |
| 24h after the second treatment | Headache | 5 | 3 | 8 |
|  | Abdominal pain | 3 | 7 | 10 |
|  | Itching | 0 | 1 | 1 |
|  | Thrill | 0 | 0 | 0 |
|  | Nausea | 0 | 1 | 1 |
|  | Vomiting | 2 | 0 | 2 |
|  | Diarrhoea | 1 | 1 | 2 |
|  | Fever (≥ 37.5 ºC) | 0 | 0 | 0 |
|  | Other | 0 | 0 | 0 |
| 3h after the third treatment | Headache | 3 | 3 | 6 |
|  | Abdominal pain | 3 | 7 | 10 |
|  | Itching | 0 | 0 | 0 |
|  | Thrill | 0 | 0 | 0 |
|  | Nausea | 0 | 0 | 0 |
|  | Vomiting | 0 | 0 | 0 |
|  | Diarrhoea | 0 | 1 | 1 |
|  | Fever (≥ 37.5 ºC) | 0 | 1 | 1 |
|  | Other | 1 | 0 | 1 |
| 24h after the third treatment | Headache | 1 | 3 | 4 |
|  | Abdominal pain | 6 | 1 | 7 |
|  | Itching | 0 | 0 | 0 |
|  | Thrill | 0 | 0 | 0 |
|  | Nausea | 0 | 1 | 1 |
|  | Vomiting | 0 | 0 | 0 |
|  | Diarrhoea | 2 | 1 | 3 |
|  | Fever (≥ 37.5 ºC) | 0 | 0 | 0 |
|  | Other | 0 | 0 | 0 |
| 48h after the third treatment | Headache | 6 | 7 | 13 |
|  | Abdominal pain | 8 | 10 | 18 |
|  | Itching | 0 | 0 | 0 |
|  | Thrill | 0 | 0 | 0 |
|  | Nausea | 0 | 3 | 3 |
|  | Vomiting | 0 | 2 | 2 |
|  | Diarrhoea | 7 | 3 | 10 |
|  | Fever (≥ 37.5 ºC) | 0 | 3 | 3 |
|  | Other | 0 | 0 | 0 |
