# Supplementary material for: Efficacy and Safety of a Single Dose versus a Multiple Dose Regimen of Mebendazole against Hookworm Infections in Children: A Randomised, Double-blind Trial
Source: eClinicalMedicine. 2018 Jul 11;1:7–13. doi: 10.1016/j.eclinm.2018.06.004 (PMC6537524; doi:10.1016/j.eclinm.2018.06.004)
Supplement: Supplementary file 2 — Supplementary material 2 [file mmc2.docx]

**Research in context**

**Evidence before this study**

We searched in PubMed for all articles published before June 1, 2017 which mentioned both “hookworm” and “mebendazole” in the abstract, with no language restrictions. Although several studies have investigated the effect of either a single or a multiple dose of mebendazole, we only identified one open-label clinical trial, which compared the effect of both the single and the multiple dose mebendazole regimen 16 years ago, prior to commencement of large-scale administration of anthelminthic drugs.

**Added value of this study**

This is the first double-blind randomised clinical trial comparing the effect of a single dose (500 mg) to a multiple dose (100 mg twice a day during three consecutive days) of mebendazole against hookworm infections in Pemba, Tanzania, a setting with high drug pressure and persistent high hookworm prevalence. The results of this study clearly showed that the multiple mebendazole dose is more effective than the single dose. Both regimens were safe with only mild adverse events being reported.

**Implications of all the available evidence**

Currently, the main control strategy against hookworm and other soil-transmitted helminths is preventive chemotherapy, which is based on the administration a single dose of either mebendazole or albendazole. Our study confirms that the curative effect of a single dose mebendazole is not sufficient for treating hookworm infections and that alternative, more effective treatments, as a multiple dose mebendazole regimens might be considered, in particular in persistent hotspot settings.
